# Iron at the interface of immunity and infection

**DOI:** 10.3389/fphar.2014.00152

**Published:** 2014-07-16

**Authors:** Manfred Nairz, David Haschka, Egon Demetz, Günter Weiss

**Affiliations:** Department of Internal Medicine VI-Infectious Diseases, Immunology, Rheumatology, Pneumology, Medical University of InnsbruckInnsbruck, Austria

**Keywords:** iron, anemia of chronic disease, bacteria, nitric oxide, interferon, hepcidin, macrophage

## Abstract

Both, mammalian cells and microbes have an essential need for iron, which is required for many metabolic processes and for microbial pathogenicity. In addition, cross-regulatory interactions between iron homeostasis and immune function are evident. Cytokines and the acute phase protein hepcidin affect iron homeostasis leading to the retention of the metal within macrophages and hypoferremia. This is considered to result from a defense mechanism of the body to limit the availability of iron for extracellular pathogens while on the other hand the reduction of circulating iron results in the development of anemia of inflammation. Opposite, iron and the erythropoiesis inducing hormone erythropoietin affect innate immune responses by influencing interferon-gamma (IFN-γ) mediated (iron) or NF-kB inducible (erythropoietin) immune effector pathways in macrophages. Thus, macrophages loaded with iron lose their ability to kill intracellular pathogens via IFN-γ mediated effector pathways such as nitric oxide (NO) formation. Accordingly, macrophages invaded by the intracellular bacterium *Salmonella enterica serovar Typhimurium* increase the expression of the iron export protein ferroportin thereby reducing the availability of iron for intramacrophage bacteria while on the other side strengthening anti-microbial macrophage effector pathways via increased formation of NO or TNF-α. In addition, certain innate resistance genes such as natural resistance associated macrophage protein function (Nramp1) or lipocalin-2 exert part of their antimicrobial activity by controlling host and/or microbial iron homeostasis. Consequently, pharmacological or dietary modification of cellular iron trafficking enhances host resistance to intracellular pathogens but may increase susceptibility to microbes in the extracellular compartment and vice versa. Thus, the control over iron homeostasis is a central battlefield in host–pathogen interplay influencing the course of an infectious disease in favor of either the mammalian host or the pathogenic invader.

## INTRODUCTION

The control over iron homeostasis is decisive in host–pathogen interaction (Schaible and Kaufmann, 2004; Nairz et al., 2010; Cassat and Skaar, 2013). This is due to the fact that iron is central for several metabolic processes for both, prokaryotic and eukaryotic cells that the metal affects microbial proliferation and pathogenicity and in addition significantly impacts on immune cell plasticity and innate immune responses. These multiple functional aspects of iron are based on its ability to transfer electrons needed during metabolic processes and to catalyze the formation of highly reactive radicals (Papanikolaou and Pantopoulos, 2005; Koskenkorva-Frank et al., 2013). The latter can act as signaling molecules but also intoxicate microbes or damage surrounding cells and tissues. Many microbes are highly dependent on a sufficient supply of iron and take up this metal by multiple and divergent pathways or steel it from iron deposition sites of the host (Winkelmann, 2002; Cassat and Skaar, 2013; Frawley et al., 2013). The activation and expression of such microbial iron acquisition systems is linked to their pathogenicity and proliferation (Rabsch et al., 1999; Schrettl et al., 2004; Crouch et al., 2008; Andrews-Polymenis et al., 2010; Cassat and Skaar, 2013). On the other hand, iron plays important roles in anti-microbial host responses, first by synergistic effects towards anti-microbial radical formation (Mastroeni et al., 2000; Esposito et al., 2003; Papanikolaou and Pantopoulos, 2005; Koskenkorva-Frank et al., 2013) but second, by directly altering immune cell proliferation and anti-microbial immune effector pathways (Nairz et al., 2010). Thus, the host immune system affects the availability of iron for microbes via the activity of cytokines, cellular proteins/peptides and hormones to gain control over pathogen proliferation and to strengthen specific immune effector pathways, a strategy for which the term “nutritional immunity has been coined.

## ALTERATION OF IRON HOMEOSTASIS DURING INFECTION AND INFLAMMATION

The most frequent and best known example visualizing the interaction between iron, immunity and infection is anemia of chronic disease (ACD) also termed as anemia of (chronic) inflammation (Cartwright, 1966; Spivak, 2002; Weiss and Goodnough, 2005). ACD is considered to be the second most frequent anemia worldwide and it develops specifically in patients suffering from chronic inflammatory diseases, such as auto-immune disorders, cancer, chronic infections or in patients undergoing dialysis (Weiss and Goodnough, 2005). The underlying pathophysiology involves mainly (i) iron retention within the monocyte/macrophage system, (ii) a blunted formation and activity of the red blood cell hormone erythropoietin (Epo), and (iii) an impaired proliferation and differentiation of erythroid progenitor cells (Weiss and Schett, 2013). All these pathophysiological pathways are driven by inflammatory signals, and the mononuclear-phagocyte system (MPS) is in the center of such alterations. Specifically, macrophages are of central importance for maintaining a sufficient supply of iron for erythropoiesis due to their role in iron re-utilization from senescent erythrocytes which are taken up by these immune cells by a process called erythrophagocytosis before being degraded leading to recovery of heme which is further processed by the enzyme heme oxygenase-1 (HO-1) yielding equal amounts of iron, biliverdin and carbon-monoxid (Delaby et al., 2005; Knutson et al., 2005; Soe-Lin et al., 2009). Under physiological conditions iron recycling by macrophages accounts for approximately 95% of the daily needs of the metal for erythropoiesis and other physiological processes (Hentze et al., 2010; Pantopoulos et al., 2012). However, during inflammation this process is blunted resulting in an impaired delivery of iron for erythropoiesis. Thereby, cytokines and acute-phase proteins affect body iron homeostasis and macrophage iron metabolism leading to an inflammation driven diversion of iron traffic which is characterized by low circulating iron concentrations and high levels of the iron storage protein ferritin , the latter reflecting iron retention in the MPS (Thomas and Thomas, 2005; Weiss and Goodnough, 2005).

**FIGURE 1:**
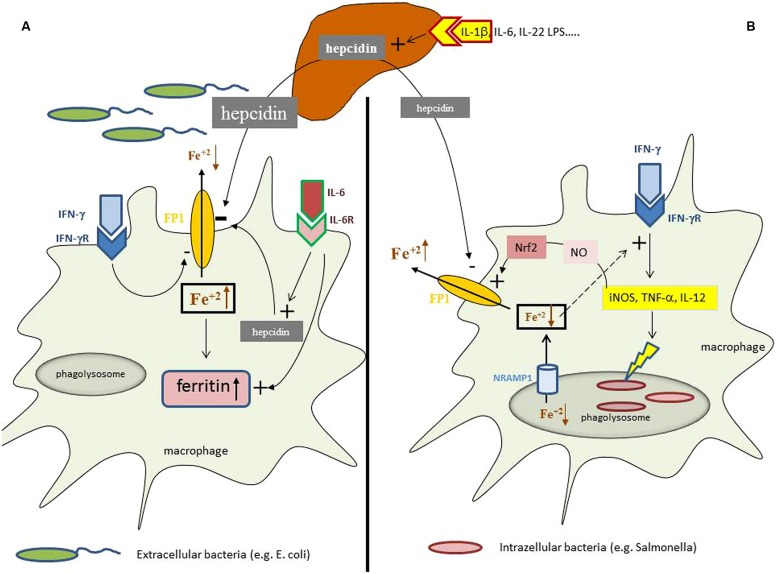
**Iron homeostasis in differently regulated depending on the primary localization of the pathogen.** During an inflammatory process cytokines and bacterial products can induce the formation of the master regulator of iron homeostasis, hepcidin, in the liver. **(A)** In case of infection with extracellular bacteria hepatocyte derived hepcidin targets ferroportin (FP1) on the cell surface and results in its degradation thereby reducing macrophage iron agrees. This effect is further supported by minute amounts of hepcidin produced by macrophages in response to interleukin-6 (IL-6) and by the action of interferon-gamma (IFN-γ) which inhibits ferroportin expression transcriptionally. This results in systemic hypoferremia and reduced iron availability for extracellular bacteria whereas the increased intracellular amounts of iron in the cell are incorporated into ferritin, the expression of which is induced by iron and cytokines, such as tumor necrosis factor-alpha (TNF-α), IL-1β, or IL-6. **(B)** While in case of an infection with intracellular bacteria hepcidin also exerts negative effects on ferroportin expression posttranslationally, this mechanisms appears to be outcompeted by massive induction of ferroportin transcription which can be traced back to activation of inducible nitric oxide synthase (iNOS) with subsequent nitric oxide (NO) formation and activation of the transcription factor Nrf2 which massively stimulate ferroportin transcription leading in summary to stimulation of iron export and reduction of intracellular iron levels. In addition, microbes residing in the phagolysome are further deprived from iron via the action of Nramp1 which pumps the metal into the cytoplasm and subsequently out of the cells by the action of ferroportin. In addition, due to the negative regulatory effect of iron on innate immune effector function and specifically IFN-γ activity, the reduction of intracellular iron levels results in stimulation of anti-microbial immune effector pathways, such as iNOS, TNF-α, or IL-12 formation which together with the reduction of cellular iron availability help to control infections with intracellular microbes.

In case of an infection, auto-immune disease or cancer immune cells are activated and produce a myriad of cytokines, some of which exerting specific effects on iron homeostasis. Cytokines such as interleukin (IL)-1, IL-6, or IL-22 as well as bacterial LPS or endoplasmic reticulum stress induce the formation of the master regulator of iron homeostasis, hepcidin, in the liver (Nemeth et al., 2003; Vecchi et al., 2009; Armitage et al., 2011). Hepcidin affects cellular iron homeostasis upon binding to the only known iron export protein ferroportin, thereby leading to ferroportin internalization and degradation which subsequently reduces cellular iron export (Nemeth et al., 2004b). As a consequence of this interaction, the absorption of iron from the diet is reduced due to hepcidin mediated reduction of ferroportin expression in enterocytes, thereby resulting in a reduction of circulating iron levels which is further aggravated by the inhibition of iron export from macrophages by the same mechanism (Nemeth et al., 2004a; Kemna et al., 2005; Roy et al., 2007; Theurl et al., 2009). Moreover, macrophages produce minute amounts of hepcidin in response to inflammatory stimuli such as IL-6 or LPS thereby blocking iron export in an autocrine fashion (Peyssonnaux et al., 2006; Theurl et al., 2008), which is meant to result from a nutritional immune strategy of the body to reduce the availability of iron for extracellular pathogens (**Figure 1**).

In parallel, cytokines exert subtle and hepcidin independent effects on the regulation of iron homeostasis on multiple levels (Weiss and Schett, 2013). First, tumor necrosis factor-alpha (TNF-α) impairs duodenal iron absorption by an as yet not fully elucidated mechanism (Johnson et al., 2004). Second, TNF-α, IL-1, IL-6, and interferon-gamma (IFN-γ) affect macrophage iron homeostasis by different avenues. They increase the uptake of transferrin and non-transferrin bound iron by modulating the expression of transferrin receptor-1 and divalent metal transporter-1, respectively (Byrd and Horwitz, 1993; Fahmy and Young, 1993; Mulero and Brock, 1999; Ludwiczek et al., 2003). In parallel, based on damaging of erythrocytes by inflammation born radicals, the half-life of erythrocytes decreases and erythrophagocytosis is stimulated. In addition, many of these cytokines as well as the anti-inflammatory cytokines IL-4, IL-10, and IL-13 promote efficient iron storage within macrophages/monocytes by increasing the expression of ferritin, both at the transcriptional and posttranscriptional level (Byrd and Horwitz, 1993; Weiss et al., 1997a; Mulero and Brock, 1999; Arosio and Levi, 2002; Tilg et al., 2002; Ludwiczek et al., 2003). Macrophage iron content is further expanded via an inhibitory effect of IFN-γ and LPS on ferroportin transcription thereby reducing cellular iron egress (Yang et al., 2002; Ludwiczek et al., 2003).

In summary the combination of these regulatory effects lead to iron retention in circulating monocytes and macrophages which present with low ferroportin expression and increased intracellular ferritin levels (Theurl et al., 2006) along with a reduction of circulating iron concentration, the diagnostic hallmark of ACD.

## INTERRELATIONSHIP BETWEEN IRON AVAILABILITY, INNATE IMMUNE FUNCTION AND CONTROL OF INFECTIONS

Considering the prevalence of ACD the question arises whether there is a specific benefit for the host upon development of ACD. Obviously, the retention of iron in the monocyte–macrophage system reduces circulating iron levels and thus the availability of this essential nutrient for extracellular microbes (**Figure 1A**). This iron withholding strategy appears to be of benefit to combat infections with circulating microbes or pathogens residing in tissues with high iron contents such as the liver (Ganz, 2009; Weinberg, 2009). In addition, the pathophysiological mechanisms underlying the development of ACD also significantly impact on the efficacy of host responses against infections. First, the reduced biological activity of Epo may ameliorate anti-bacterial immune responses. This is based on the fact that the major erythropoiesis stimulating hormone Epo also exerts effects on cells and tissues outside the bone marrow, and such effects are transduced by a heterodimeric receptor which differs from the receptor expressed on erythroid progenitor cells (Brines and Cerami, 2012). Thereby, Epo inhibits pro-inflammatory immune effector pathways in macrophages via inhibition of NF-kB activation which results in reduced expression of inducible nitric oxide synthase (iNOS), TNF-α, IL-6, and IL-12 by inflammatory macrophages and an impaired immune control of infections with bacteria such as *Salmonella enterica serovar typhimurium* (*S. typhimurium*; Nairz et al., 2011). Accordingly, the reduced Epo activity observed in ACD results in a sustained pro-inflammatory immune response and an improved control of *S. typhimurium* septicemia. Second, iron exerts multiple effects on immune effector functions. This is on the one hand based on the role of iron for the differentiation and proliferation of immune cells, including antigen presenting cells and lymphocytes (reviewed by Oppenheimer, 2001; Weiss, 2002; Cairo et al., 2011). Moreover, iron affects anti-microbial immune function of macrophages via inhibition of IFN-γ inducible effector pathways (Weiss et al., 1992; Oexle et al., 2003). Thus, macrophages loaded with iron lose their ability to clear infections with intracellular pathogens, such as *Salmonella*, *Mycobacteria*, *Chlamydia*, *Candida*, or *Legionella* (Mencacci et al., 1997; Chlosta et al., 2006; Nairz et al., 2007; Paradkar et al., 2008; Botella et al., 2012; Bellmann-Weiler et al., 2013) whereas reduction of iron levels or addition of iron chelators can improve infection control by withholding iron from microbes and by increasing anti-microbial immune effector functions. Thereby iron chelators antagonize a direct inhibitory effect of iron toward the expression of iNOS (Weiss et al., 1994; Melillo et al., 1997; Dlaska and Weiss, 1999). iNOS catalyzed high output formation of the labile radical NO by macrophages (Nathan and Shiloh, 2000; Bogdan, 2001) which exerts direct anti-microbial effector functions. This pathway appears to be partly responsible for the beneficial clinical effects of iron chelators observed in several experimental infection systems (Mencacci et al., 1997; Fritsche et al., 2001; Ibrahim et al., 2007; Bellmann-Weiler et al., 2013; Nairz et al., 2013). Along this line children suffering from cerebral malaria benefited from the addition of the iron chelator desferrioxamine to a standard anti-malaria treatment, as reflected by faster recurrence from coma and clearance of plasmodia, although this did not translate into a survival benefit (Gordeuk et al., 1992; Mabeza et al., 1999), although anti-plasmodial innate immune responses were positively affected by iron chelation *in vivo* (Thuma et al., 1996; Weiss et al., 1997b). Of interest, via its inhibitory potential on IFN-γ iron affects T-helper (TH) cell differentiation favoring the expansion of TH-2 cells which produce a number of macrophage-deactivating cytokines such as IL-4 or IL-13 (Weiss, 2002). Accordingly, iron overload and iron chelation have been shown to modulate the TH-1/TH-2 in mice exposed to different microbes (Mencacci et al., 1997; Ibrahim et al., 2007; Nairz et al., 2013). However, a recent clinical trial in patients after allogenic bone marrow transplantation who received the iron chelator desferrasirox in an attempt to improve the control of mucormycosis, a devastating invasive fungal infection in severely immuno-compromised patients, failed to show a clinical benefit (Spellberg et al., 2012). In contrast, the mortality rate was even higher in those subjects receiving desferrasirox and the reason for that has been elusive thus far. Hypothetically this may be partly related to an effect of desferrasirox on innate immune function, which could have been resulted in aggravation of graft versus host disease, a notion which should be verified at least retrospectively.

However, from all the evidence listed above it appears obvious that ACD develops from the endeavor of the body to withhold iron from invading, extracellular pathogens and to strengthen at the same time anti-microbial immune responses (**Figure 1A**).

Accordingly, clinical trials which were performed to supplement iron to children in developing countries based on the notion that iron deficiency is associated with growth and mental retardation produced unpredicted results (Schumann et al., 2007). These studies demonstrated that iron supplementation resulted in higher incidence of or higher mortality from infections such as malaria, diarrhea or bacterial meningitis (Sazawal et al., 2006; Soofi et al., 2013). The pathways underlying these devastating outcomes remain elusive thus far. However, they may be linked to iron mediated modulation of anti-microbial immune defense mechanisms or traced back to increased availability of the metal for pathogens in the setting of subclinical parasitemia or bacteremia. The cause-effective association between iron availability and the clinical course of malaria has been well established by several recent studies.

Those studies provided evidence for a reduced risk of malaria in general and severe malaria in iron deficient pregnant women and children (Kabyemela et al., 2008; Gwamaka et al., 2012; Jonker et al., 2013) whereas other studies found interesting associations between iron delivery for erythropoiesis, circulating hepcidin levels and the prevalence of malaria in tropical countries (de Mast et al., 2010; Prentice et al., 2012; Atkinson et al., 2014).

Apart from plasmodia, the availability of iron is of importance for other parasitic infection such as Leishmaniosis or Trypanosomiasis. The pathogenicity of *Leishmania* is linked to the expression of different microbial iron acquisition molecules and a sufficient supply of iron (Mittra et al., 2013). Accordingly, inhibition of cellular iron export can promote *Leishmania* proliferation (Ben-Othman et al., 2014) whereas over-expression of ferroportin with subsequent limitation of intracellular iron availability limits intracellular leishmanial growth (Rafiee et al., 2014). In addition, drugs such as quercetin exert their anti-microbial activity against *Leishmania donovani* by interfering with microbial iron acquisition (Sen et al., 2008). Of note, *L. donovani* manipulates macrophage iron homeostasis to increase its own iron supply (Das et al., 2009). However, via its radical promoting capacity iron can also exert protective effects in *Leishmania* infection by strengthening radical dependent host response as shown in a mouse model of *Leishmania infantum* infection (Vale-Costa et al., 2013). Similarly, *Trypanosma* species are also highly dependent on a sufficient supply of iron which depending on the subtype is acquired by classical sources (transferrin) or taken up via ZIP family transporters (Taylor and Kelly, 2010). Given the central role of iron for the pathogenesis of trypansoma infections, the host aims at limiting iron availability to these parasites which results in alterations of macrophage iron homeostasis along with the development of anemia (Stijlemans et al., 2008, 2010). Of interest, the induction of the Nrf2 pathway exerted protective effects in a model of *Trypanosma cruci* infection in mice which was counterbalanced by the addition of iron sulfate (Paiva et al., 2012), suggesting that Nrf2 exerted its anti-trypanosmal activity by increasing the expression of ferroportin (Nairz et al., 2013).

## METABOLIC IRON RESPONSES AND IMMUNE CONTROL OF INTRACELLULAR PATHOGENS

Although still insufficiently understood, it is hypothesized that the presence of extracellular pathogens in the circulation induces iron restriction in the monocyte/macrophage system via the action of hepcidin and several cytokines, whereas the metabolic responses to intracellular microbes appear to be different (**Figure 1**). Macrophages infected with *S. typhimurium* increase the expression of ferroportin and stimulate iron export (Nairz et al., 2007). This leads to a reduced availability of iron for intracellular bacteria along with an activation of anti-microbial immune effector mechanisms due to counter-balancing the negative regulatory effects of iron on IFN-γ inducible immune pathways (Oexle et al., 2003; Nairz et al., 2007; Weiss and Schett, 2013). Similar observations have been made with other intracellular pathogens such as Chlamydia spp. or Legionella (Chlosta et al., 2006; Paradkar et al., 2008; Bellmann-Weiler et al., 2010) Of interest, ferroportin is also expressed in mycobacteria containing phagosomes where it pumps iron into the cytoplasma (Van Zandt et al., 2008), a pathway which limits the availability of this essential nutrient for this bacterium (Schnappinger et al., 2003; Olakanmi et al., 2013). This indicates that the stimulation of iron export via ferroportin is an efficient defense strategy against infection with intracellular microbes by limiting their access to iron and by strengthening anti-microbial immune effector pathways (Nairz et al., 2010).

Recent evidence suggest, that the intracellular bacterium *Salmonella typhimurium* can counteract these metabolic immune defense strategies by inducing hepcidin expression in hepatocytes via activation of estrogen related receptor (ERR)-gamma, thereby resulting in hypoferremia and macrophage iron retention with a subsequently increased availability of the metal for intracellular *Salmonella* (Kim et al., 2014). A reverse agonist of this pathway counter-acting ERR-gamma and hepcidin mediated macrophage iron retention led to an improved control of *Salmonella* infection (Kim et al., 2014). This is in a line with previous observations demonstrating that modulation of the hepcidin/ferroportin axis impacts on intracellular proliferation of *Salmonella* and the course of infection in mice (Nairz et al., 2009b; Wang et al., 2009).

Importantly, several innate resistance mechanisms exert at least part of their anti-microbial activity via restriction of iron to microbes.

### NO

High output formation of NO by immune cells is of central importance for immune control of infections and cancer (Nathan and Shiloh, 2000; Bogdan, 2001). An interaction of NO with iron homeostasis has been well documented based on the fact that (i) NO has a high affinity for iron and that many of the cytotoxic effects of NO are based on targeting of central iron sulfur clusters in enzymes by the labile radical (Nathan and Shiloh, 2000; Bogdan, 2001), that (ii) NO controls intracellular iron trafficking by regulating the binding affinity of iron regulatory proteins (IRP) to specific RNA stem loop structures within the non-coding regions of critical iron metabolism genes (Drapier et al., 1993; Weiss et al., 1993; Pantopoulos et al., 1996), and finally, that (iii) cellular iron content controls NO expression by regulating iNOS transcription (Weiss et al., 1994; Melillo et al., 1997; Dlaska and Weiss, 1999). Recent evidence now suggests that part of the anti-microbial effect of NO can be attributed to a regulatory activity of the radical on iron homeostasis. Thereby, NO activates the binding affinity of the transcription factor Nrf-2 to the ferroportin promoter, resulting in increased ferroportin expression and iron export (Nairz et al., 2013). This results in a reduced availability of iron for intra-macrophage bacteria and a strengthening of anti-microbial, IFN-γ driven immune responses. Of note, iNOS^-/-^ mice present with macrophage iron overload and their resistance against infection with *Salmonella* can be increased upon application of a membrane permeable iron chelator such as desferrasirox (Nairz et al., 2013).

### Nramp1

The natural resistance macrophage protein 1 (Nramp1 or Slc11a1) has been characterized as a late phagosomal protein conferring resistance to infection with *Salmonella*, *Mycobacteria*, and *Leishmania* (Blackwell et al., 2000; Forbes and Gros, 2001). Nramp1 exerts its protective effect against such infections via acidification of the microbe containing phagosome, but also by altering the cellular distribution of divalent metals such as zinc, manganese or iron which all play decisive role in host–pathogen interaction (Hood and Skaar, 2012; Diaz-Ochoa et al., 2014). In addition, the functional expression of Nramp1 strengthens anti-microbial immune effector pathways such as the formation of TNF-α or NO (Barton et al., 1995; Fritsche et al., 2003). These effects can be traced back to prolonged activity of pro-inflammatory signaling pathways and inhibition of the expression of anti-inflammatory cytokines, such as IL-10 (Fritsche et al., 2008). Evidence accumulates, that Nramp1pumps iron out of macrophages thereby reducing iron levels in the cytoplasma and within the phagolysosome rendering the metal less available for intracellular bacteria (Barton et al., 1999; Zwilling et al., 1999; Kuhn et al., 2001; Nairz et al., 2009a; Sohn et al., 2011). As a further consequence of intracellular iron depletion, pro-inflammatory immune effector pathways are augmented. One of these Nramp1-inducible responses is the increased formation of lipocalin-2 (Lcn2 or NGAL), which blocks another source of iron for bacteria (Fritsche et al., 2012).

### LIPOCALIN-2

Lcn2 is produced by several cells in the body including neutrophils and macrophages (Chakraborty et al., 2012). Among many other functions it binds the bacterial siderophore enterobactin, which is produced by Gram-negative bacteria such as *Escherichia coli*, *Klebsiella*, or *Salmonella* spp. to scavenge iron and to redeliver the metal to the microbe where it is taken up via specific receptors. Mice expressing Lcn2 are more resistant to infections with such Gram-negative bacteria as compared to Lcn2^-/-^ littermates which is cause-effectively due to the bacterial iron withholding capacity of Lcn2 (Flo et al., 2004; Berger et al., 2006). Of note, the varying dependence of bacteria from siderophore mediated iron uptake can cause a growth advantage of certain bacteria among others. It has recently been demonstrated that the pro-biotic bacterium *E. coli* Nissle controls the growth of pathogenic *Salmonella* in the intestine by over-coming iron restriction by Lcn2 (Deriu et al., 2013). Accordingly, resistance of *Salmonella* to Lcn2 mediated iron restriction can cause a growth advantage of this pathogen in the gut (Raffatellu et al., 2009). It is of interest, that Lcn2 not only affects microbial iron delivery but also host iron homeostasis. This is most likely due to binding of a recently identified mammalian siderophore by Lcn2 which then can shuttle iron across cellular membranes (Bao et al., 2010; Devireddy et al., 2010). Of note, recent evidence also suggests that the mammalian siderophore 2,5-DHBA can be utilized by Gram-negative bacteria as a source of iron and that macrophages reduce the expression of 2,5-DHBA when exposed to LPS or Gram negative bacteria which is also considered to be part of the “iron withholding” defense network of innate immune cells (Liu et al., 2014).

Lcn2 appears to be of importance to mount alterations of iron host homeostasis on the cellular and systemic level thereby contributing to hypoferremia and intracellular iron depletion in systemic *Salmonella* infection (Nairz et al., 2009b; Srinivasan et al., 2012). An increased expression of Lcn2 by macrophages along with reduced intramacrophage iron content and impaired bone morphogenetic signaling may be largely responsible for the reduced susceptibility of Hfe^-/-^ mice, a model for hereditary hemochromatosis, against infection with the intracellular bacteria *Salmonella* and *Mycobacteria* (Olakanmi et al., 2007; Corradini et al., 2009; Nairz et al., 2009b), which may also be a reason of the high penetrance of this genes in people of Northern and Western European origin (Pietrangelo, 2004). Accordingly, Lcn2 expression positively affects the course of infection with other intracellular pathogens, even if they do not express siderophores such as Chlamydia (Bellmann-Weiler et al., 2013), whereas cellular iron export and increased delivery of the metal to the extracellular space can be detrimental as observed in a model of pneumococcal infection, where Lcn2 expression by neutrophils resulted in increased mortality of mice (Warszawska et al., 2013).

This again provides evidence that contrasting pathways for the regulation of iron homeostasis according to infection with either intracellular or extracellular pathogens exist (Chan et al., 2009; Warszawska et al., 2013; Fang and Weiss, 2014) which are still insufficiently understood (**Figure 1**).

Owing to the importance of these pathways for immune defense against infection with intracellular pathogens the central TH-1 cytokine IFN-γ exerts part of its anti-microbial effects by stimulating immune responses which restrict the availability of iron for microbes. IFN-γ induces the expression of Nramp1, Lcn2, and NO thereby reducing intracellular iron content in macrophages and limiting the growth of bacteria such as *Salmonella* (Gruenheid et al., 1997; Fritsche et al., 2008; Nairz et al., 2008, 2009a; Andrews-Polymenis et al., 2010; Nairz et al., 2013). Thus, IFN-γ is central for host response in *Salmonella* infection based on these nutritional iron effects but also due to its ability to induce a myriad of anti-bacterial effector mechanisms in macrophages including oxygen and nitrogen radical formation or maturation of the bacterial containing phagosome (Mastroeni et al., 2000; McCollister et al., 2005; Henard and Vazquez-Torres, 2011). Of note, neutrophils have recently been shown to produce IFN-γ to further stimulate anti-bacterial immune pathways (Spees et al., 2014) whereas *S. typhimurium* has been shown to impair the proliferation of CD4 cells, a major source of IFN-γ (Atif et al., 2014).

## TARGETING IRON HOMEOSTASIS IN INFECTIOUS DISEASE-TO WALK A TIGHTROPE

While iron supplementation in eras with a higher burden of infectious diseases caused detrimental effects toward the risk of malaria or invasive bacterial infections (Sazawal et al., 2006; Soofi et al., 2013), iron supplementation in HIV infected patients resulted in an impaired control of malaria but also in a beneficial or at least neutral course of HIV infection at as reflected by CD4+-cell counts or progression of the disease which was partly dependent on the degree of anemia and base-line iron status (Esan et al., 2013; Prentice et al., 2013; Zlotkin et al., 2013). However, HIV infection is often associated with reactivation of *Mycobacterium tuberculosis* infection, and iron has been shown to be an essential nutrient for such bacteria which goes along with the observation that iron loading is associated with an increased risk for tuberculosis and an adverse clinical course of this infection (Moyo et al., 1997; Gangaidzo et al., 2001; Schaible and Kaufmann, 2004). Accordingly, iron supplementation in subjects with latent tuberculosis and an impaired immune control, e.g., on the basis of HIV infection, is hazardous (McDermid et al., 2013). Of interest, a recent study suggested that both, iron deficiency and iron loading, are associated with an adverse clinical course of tuberculosis, both in HIV positive and negative subjects (Isanaka et al., 2012a,b). This may relate to the divergent effects of iron on the immune system, on the one hand iron is a prerequisite for immune cell proliferation and differentiation (Thorson et al., 1991; Weiss, 2002; Porto and De Sousa, 2007), and a catalyzer for the formation of anti-microbial radicals (Mastroeni et al., 2000; Papanikolaou and Pantopoulos, 2005), whereas on the other hand it impacts on innate immune effector functions (Weiss and Schett, 2013) and positively affects microbial proliferation (Schaible and Kaufmann, 2004; Weinberg, 2009; Nairz et al., 2010; Cassat and Skaar, 2013; **Figure 1B**). Thus, a certain balance of iron, not too less and not too much, is needed to strengthen immune responses to successfully combat infections (Drakesmith and Prentice, 2012). Of note, alterations of iron homeostasis have been shown to affect the composition of the human microbiome and may thereby alter the proliferation of pathogenic bacteria (Deriu et al., 2013).

Accordingly, pharmacological concepts to modify iron homeostasis and iron trafficking in an attempt to combat infection have always to keep in mind that a positive effect on one infection may have devastating effects on the course of another infection as seen in models of malaria where tolerance induction via overexpression of HO improves the course of malaria but increases the risk for bacterial infections such as Salmonellosis (Portugal et al., 2011; Gozzelino et al., 2012). Opposite, reduction of intracellular/macrophage iron levels upon therapeutic application of the calcium-antagonists nifedipine resulted in significantly improved survival of mice with *S. typhimurium* (Mair et al., 2011). Strictly speaking, any therapeutic strategy, e.g., iron chelation, iron mobilization, hepcidin or anti-hepcidin pharmacological approaches, which help to control the course of one, e.g., intracellular infection, may increase the availability of iron for a pathogen residing in a different compartment, e.g., in the extracellular space, along with the unpredictable effects on host immune response (Nairz et al., 2010; Drakesmith and Prentice, 2012). Thus, major research efforts must be undertaken to better understand the diverse roles of iron in infection and in immune control of infections. Specifically, we also need to address the metabolic alterations of iron homeostasis in response to different pathogens, not only in terms of their primary tissue localization but also in relation to their needs for iron and the immune mechanisms being involved in their control.

## Conflict of Interest Statement

The authors declare that the research was conducted in the absence of any commercial or financial relationships that could be construed as a potential conflict of interest.
